# piggyBac Transposon Somatic Mutagenesis with an Activated Reporter and Tracker (PB-SMART) for Genetic Screens in Mice

**DOI:** 10.1371/journal.pone.0026650

**Published:** 2011-10-21

**Authors:** Sean F. Landrette, Jonathan C. Cornett, Thomas K. Ni, Marcus W. Bosenberg, Tian Xu

**Affiliations:** 1 Department of Genetics, Yale University School of Medicine, Boyer Center for Molecular Medicine, Howard Hughes Medical Institute, New Haven, Connecticut, United States of America; 2 Departments of Dermatology and Pathology, Yale University School of Medicine, New Haven, Connecticut, United States of America; 3 School of Life Science, Fudan-Yale Center for Biomedical Research, Institute of Developmental Biology and Molecular Medicine, Fudan University, Shanghai, China; Duke University Medical Center, United States of America

## Abstract

Somatic forward genetic screens have the power to interrogate thousands of genes in a single animal. Retroviral and transposon mutagenesis systems in mice have been designed and deployed in somatic tissues for surveying hematopoietic and solid tumor formation. In the context of cancer, the ability to visually mark mutant cells would present tremendous advantages for identifying tumor formation, monitoring tumor growth over time, and tracking tumor infiltrations and metastases into wild-type tissues. Furthermore, locating mutant clones is a prerequisite for screening and analyzing most other somatic phenotypes. For this purpose, we developed a system using the *piggyBac* (PB) transposon for somatic mutagenesis with an activated reporter and tracker, called PB-SMART. The PB-SMART mouse genetic screening system can simultaneously induce somatic mutations and mark mutated cells using bioluminescence or fluorescence. The marking of mutant cells enable analyses that are not possible with current somatic mutagenesis systems, such as tracking cell proliferation and tumor growth, detecting tumor cell infiltrations, and reporting tissue mutagenesis levels by a simple *ex vivo* visual readout. We demonstrate that PB-SMART is highly mutagenic, capable of tumor induction with low copy transposons, which facilitates the mapping and identification of causative insertions. We further integrated a conditional transposase with the PB-SMART system, permitting tissue-specific mutagenesis with a single cross to any available Cre line. Targeting the germline, the system could also be used to conduct F1 screens. With these features, PB-SMART provides an integrated platform for individual investigators to harness the power of somatic mutagenesis and phenotypic screens to decipher the genetic basis of mammalian biology and disease.

## Introduction

Transposon insertional mutagenesis (TIM) is a powerful tool for inducing and identifying mutations of interest and has been utilized with great effect in many organisms, from the bacterium to the fruit fly *Drosophila melanogaster* [1], [2]. The *Sleeping Beauty* (SB) and *piggyBac* (PB) DNA transposons have been recently developed for TIM in mice and human cells [3], [4], [5]. As one of the first applications of TIM in mammals, SB and PB have been used to identify tumor-promoting genes for multiple tumor types in mice [6]–[13]. In addition, SB transposase has been activated by tissue-specific Cre expression for the successful induction of colorectal adenocarcinoma, hepatocellular carcinoma, and B-cell lymphoma [10], [11], [13]. The successful induction of tumors by SB and PB indicates that a more versatile somatic TIM system can be established for broad application.

Somatic genetic screening requires animal breeding and significant lengths of time for manifestation of phenotypes such as tumorigenesis. Thus, it represents substantial investments of cost and time for mammals. Although tumor induction by SB TIM has been successful in multiple tissues, tumors in a number of other tissues have yet to be found despite employing the same mutator transposons and whole-body mutagenesis approach. This highlights the importance of determining the feasibility of TIM for the tissue of interest. Although one could molecularly determine mutagenesis efficiency before the manifestation of phenotype, it is not desirable to sacrifice valuable experimental animals during the course of the screen. It would be ideal to have a method to easily determine whether TIM in a targeted tissue is feasible at the outset of the screen without sacrificing animals.

Moreover, while tumors are readily identifiable, pre-tumorous lesions and metastatic clones are difficult to locate without visible markers. Furthermore, marking mutant clones is a prerequisite for screening and analyzing most other somatic phenotypes. Visibly marking mutant somatic clones has been employed in *Drosophila*, zebrafish, and mice and demonstrated to have tremendous utility in analyzing a variety of processes including cell proliferation and migration, neurobiology and other clonal behaviors *in vivo* [14]–[17]. Thus, a somatic TIM system that can be used to screen for phenotypes other than tumor formation must incorporate the ability to track mutagenized cells.

Somatic mutagenesis screening has the genetic power to conduct genome-wide interrogation with a small number of animals. A highly efficient somatic TIM system would allow genetic screens to be performed with low copy numbers of transposon, thus allowing easy identification of causative mutations. Finally, it is optimal that the genetic elements for the features described above to be integrated such that somatic TIM can be conducted with one generation of breeding.

The PB transposon system has been shown to be highly efficient in mammals [3]. Its distinct properties of precise excision [3], [18], large payload capacity [3], and genome-wide insertion tendency [3], [12] offer a unique opportunity to create a somatic TIM system with broad utility. Here, we describe a method for PB somatic mutagenesis with an activated reporter and tracker in mice (PB-SMART). In this system, mobilization of the PB mutator transposon activates luciferase for the *ex vivo* reporting of mutagenesis activity levels, providing feasibility assessment at the outset of a genetic screen. The activation of luciferase or a red fluorescent protein also featured in this system further enables visual tracking of mutated cells. The integration of the mutagenesis reporter, mutant cell tracker, and mutagenic transposon into a single transgene greatly simplifies genetic breeding. Further incorporation of Cre-inducible PB transposase (PBase) transgenes allows tissue-specific somatic TIM screens to be conducted with one genetic cross. Finally, we show that PB-SMART can induce somatic phenotypes, track clonal behavior of mutated cells, and allow easy identification of causative insertions. Thus, PB-SMART provides a powerful means for individual investigators to decipher the genetic basis of mammalian biology and disease.

## Results

### Generating a PB mutator transposon for multiple genomic contexts

**Figure 1:**
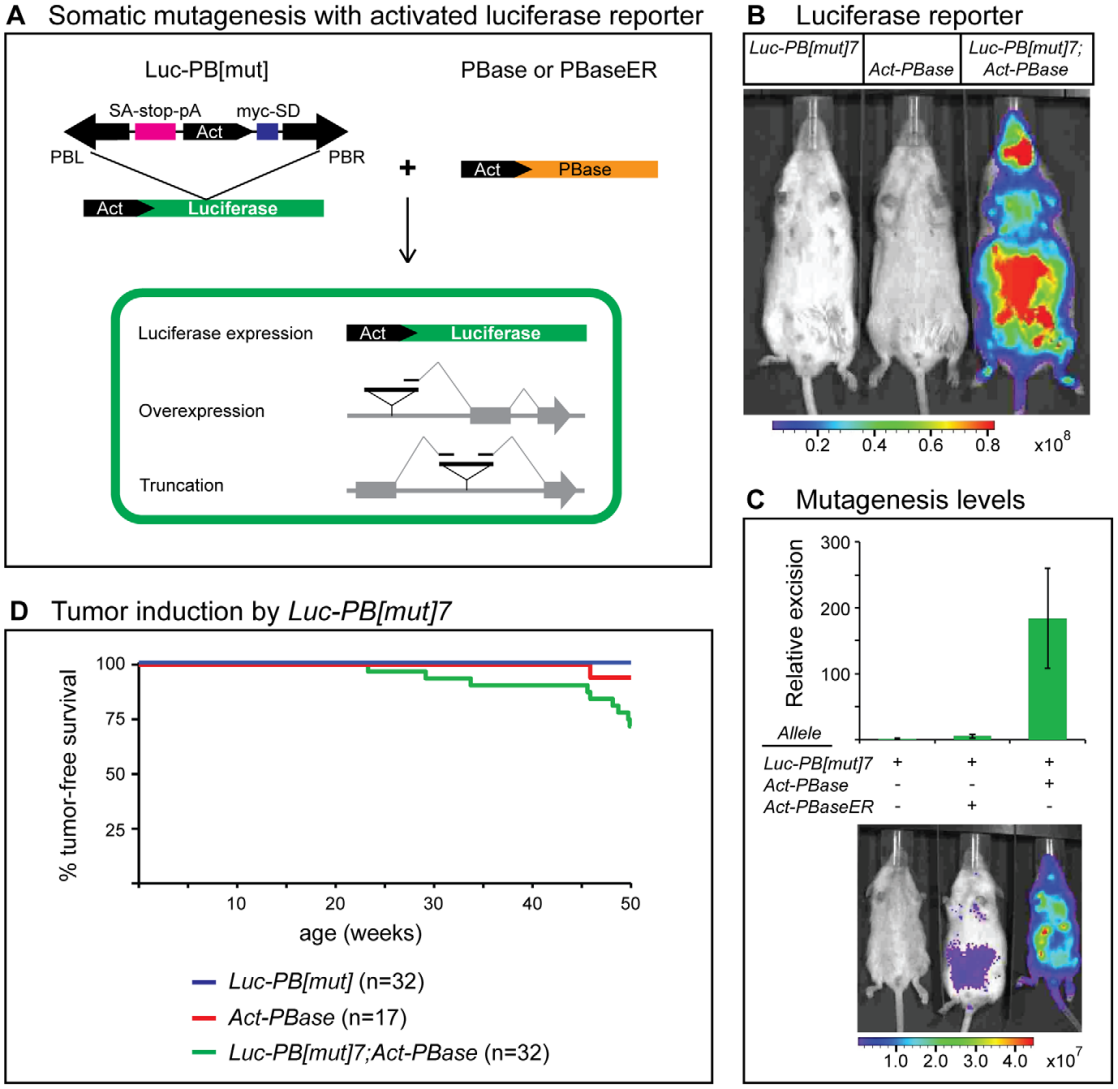
Simultaneously inducing mutations and reporting mutagenesis activity levels with a PB transposon system. (**A**) The Actin promoter (black pointed box) and myc-splice donor sequence (blue box) are engineered in the *PB[mut]* transposon to ectopically express downstream genes or partial transcripts. A splice acceptor and transcriptional termination sequences (pink box) truncate transcripts initiating upstream of the transposon insertion site. *PB[mut]* is inserted within an internal TTAA sequence of the luciferase gene to create *Luc-PB[mut]*. Transposase excises *PB[mut]* from the luciferase gene, restoring the luciferase expression and thus labeling cells. (**B**) By *ex vivo* luciferase imaging, PB mutagenesis occurs in *Luc-PB[mut]7;Act-PBase* mice (right) but not in single transgenic littermates (left and middle). Units, photons·s^−1^·cm^−2^·sr^−1^.(**C**) Quantitative PCR analysis of transposon excision in the *Luc-PB[mut]7* line demonstrates a 30-fold increase in transposition activity between *Act-PBase* and *Act-PBaseER* (top). The difference in mutagenesis activity levels driven by *Act-PBaseER* (middle) and *Act-PBase* lines (right) can be differentiated by the luciferase PB reporter. Units, photons·s^−1^·cm^−2^·sr^−1^. (**D**) Tumor-free survival curves over the span of 50 weeks show that *Luc-PB[mut]7;Act-PBase*(*Cre*) mice succumb to tumors while *Luc-PB[mut]7*(*Cre*) and *Act-PBase*(*Cre*) littermates remain relatively tumor-free.

PB has previously been found to integrate at high frequency near or within genes [3]. To efficiently induce somatic mutations, we generated a PB mutator transposon (Figure 1A, *PB[mut]*) to induce ectopic gene expression in multiple genomic contexts. The CMV early enhancer/chicken *β-actin* promoter was cloned between the PB transposon arms to induce overexpression of genes downstream of a transposon insertion site. A myc epitope in all three reading frames and a splice donor were placed after the Actin promoter such that transcription and translation initiation can incorporate the myc tag sequence prior to splicing into endogenous exons. The CMV enhancer can also act as a bidirectional enhancer to upregulate genes. Additionally, in the event that *PB[mut]* inserts into an intron, an N-terminally truncated product can be ectopically induced. Insertions into introns can also produce C-terminally truncated gene products initiated by the endogenous promoter. A splice acceptor followed by a sequence with stop codons in all three frames and a poly(A) signal was added for this purpose. Expression of truncated genes may produce dominant active and dominant negative proteins. Thus, by design, the *PB[mut]* transposon can induce the ectopic expression of full-length or truncated endogenous genes in many different genomic contexts (Figure 1A). Indeed, *PB[mut]* can induce ectopic full-length and truncated gene expression in PB-SMART screens (see below).

### PB somatic mutagenesis with an activated luciferase reporter

In order to develop a somatic mutagenesis system that can induce mutations while simultaneously and non-invasively (*ex vivo*) reporting mutagenesis levels in tissues, we engineered a strategy to bioluminescently mark cells in which transposon mobilization has occurred. We took advantage of the highly sensitive luciferase reporter gene [19] by inserting *PB[mut]* at a TTAA tetranucleotide target site within the coding sequence (Figure 1A, *Luc-PB[mut]*), thereby preventing luciferase expression from an upstream Actin promoter. During mutagenesis, active PB transposase (PBase) catalyzes the precise excision of the PB mutator transposon and a full-length luciferase product is reconstituted (Figure 1A). Five *Luc-PB[mut]* transgenic lines were established and their ability to report PBase activity was tested by crossing to our previously described *Act-PBase* line [3]. The *Luc-PB[mut]7* line was identified as the most robust reporter for PB mutagenesis activity that can be easily assayed by *ex vivo* imaging (Figure 1B). *Luc-PB[mut]7* behaves as a faithful reporter for PBase activity, since it does not produce any luciferase signal in mice lacking PBase (Figure 1B).

To examine whether *Luc-PB[mut]7* can differentiate between varying levels of PBase activity, we generated transgenic mice expressing a PBase-Estrogen Receptor fusion transgene (*Act-PBaseER*). In the inactive state, the PBaseER protein is sequestered in the cytoplasm. Stimulation with tamoxifen shuttles PBaseER to the nucleus and activates its ability to drive PB transposition (Figure S1). *In vivo*, luciferase signal was not observed in newly weaned, untreated *Luc-PB[mut]7;Act-PBaseER* transgenic mice. However, transgenic *Act-PBaseER* can mobilize PB upon tamoxifen administration (Figure 1C) and luciferase signal can be detected one week after treatment, providing a tool for conditional PB TIM. Quantitative PCR measuring *PB[mut]* excision revealed that PBaseER activity levels were significantly lower than those of *Act-PBase* (Figure 1C). These results were corroborated by the luciferase mutagenesis reporter signal from *ex vivo* imaging (Figure 1C). Furthermore, we sometimes observed leaky luciferase signal in older, untreated mice housed together with tamoxifen-treated mice (data not shown). This signal, however, is at least an order of magnitude lower than signal from treated mice as measured by *ex vivo* imaging (data not shown). The ability to differentiate between differing levels of luciferase signal indicates that *Luc-PB[mut]7* is a sensitive reporter for PBase activity.

By real-time PCR analysis, the *Luc-PB[mut]7* transgenic line was found to contain seven copies of the mutagenic transposon. When crossed to *Act-PBase* mice, the number of live-born double transgenic pups was 93% of the expected number (n>100). Since mutagenesis in *Luc-PB[mut]7;Act-PBase* mice was observed in all tissues as reported by luciferase, we addressed whether this low copy line is sufficient to induce different phenotypes when mutagenesis is targeted in all tissues or specific organs. We found *Luc-PB[mut]7* can induce tumor formation as well as other phenotypes including cellular features (*e.g.*, cell infiltration and clonal expansion) and morphological defects (*e.g.*, alopecia) (also see below).

We found that body-wide PB-SMART can induce tumor formation in non-sensitized backgrounds. We observed that *Luc-PB[mut]7;Act-PBase* and *Luc-PB[mut]7;Act-PBase;Cre* mice developed tumors while conducting sensitized screens. Nine out of 32 of these *Luc-PB[mut]7;Act-PBase*(*Cre*) mice developed tumors within 50 weeks while only one out of seventeen *Act-PBase*(*Cre*) and zero out of 32 *Luc-PB[mut]*(*Cre*) controls developed a tumor (Figure 1D). We have collected additional tumors from cohorts that have not reached 50 weeks and other non-sensitized cohorts containing floxed latent alleles of *Pten* loss or *Braf* activation [20], [21]. Across these cohorts, we have observed similar tumor types including kidney and pancreatic tumors, lung adenocarcinoma, hepatocellular carcinomas, soft tissue sarcomas such as angiosarcoma and small round blue cell (SRBC) tumors, and skin tumors such as squamous cell carcinoma, sebaceous cancer, and melanoma. These results confirm that our low copy PB-SMART system can efficiently induce tumor-promoting mutations in a wide variety of tissues.

We also found that the low copy mutagenic transposon in the PB-SMART system allows for easy identification of potential causative insertional mutations. Transposon insertions from tumors were mapped by linker-mediated PCR (LM-PCR). On average, across 45 tumors analyzed, 6.1 PB insertions were mapped. Notably, greater than 64% of all insertions were recovered in introns, exons, or within ten kilobase pairs (kb) of either known or predicted genes (Table 1). Given the propensity of *Luc-PB[mut]* for inserting into or near genes in these tumors, we analyzed whether the mutator transposon was oriented along or opposite the coding direction of inserted genes. Because our mutator transposon is designed to overexpress full-length genes or truncated genes, we expected that in tumors, insertions oriented along the coding direction of nearby or affected genes would be enriched versus insertional orientation by random chance. Indeed, over 64% of intron insertions were oriented along the coding direction of the affected genes, and almost 60% of insertions within 10 kb upstream of genes were oriented toward the coding direction of the nearby genes (Table 2). Meanwhile, insertions within 10 kb downstream of nearby genes displayed less bias for mutator orientation (Table 2). These data imply that the PB mutator transposon is indeed upregulating genes important for tumorigenesis.

**Table 1:** Insertional frequency in tumors induced by *Luc-PB[mut]7* and *PB[mut-RFP]7*.

PB insertions in	% of total hits
Genes (including ±10 kb)	64.8
known genes	57.5
predicted genes	7.3
Exons	1.8
Introns	48.0
5′ regulatory sequences (≤10 kb)	9.9
3′ regulatory sequences (≤10 kb)	4.8

**Table 2:** Mutator orientation in tumors induced by *Luc-PB[mut]7* and *PB[mut-RFP]7*.

PB insertions in	% of hits in coding direction
Exons	60.0
Introns	64.1
5′ regulatory sequences (≤10 kb)	59.3
3′ regulatory sequences (≤10 kb)	53.8

**Figure 2:**
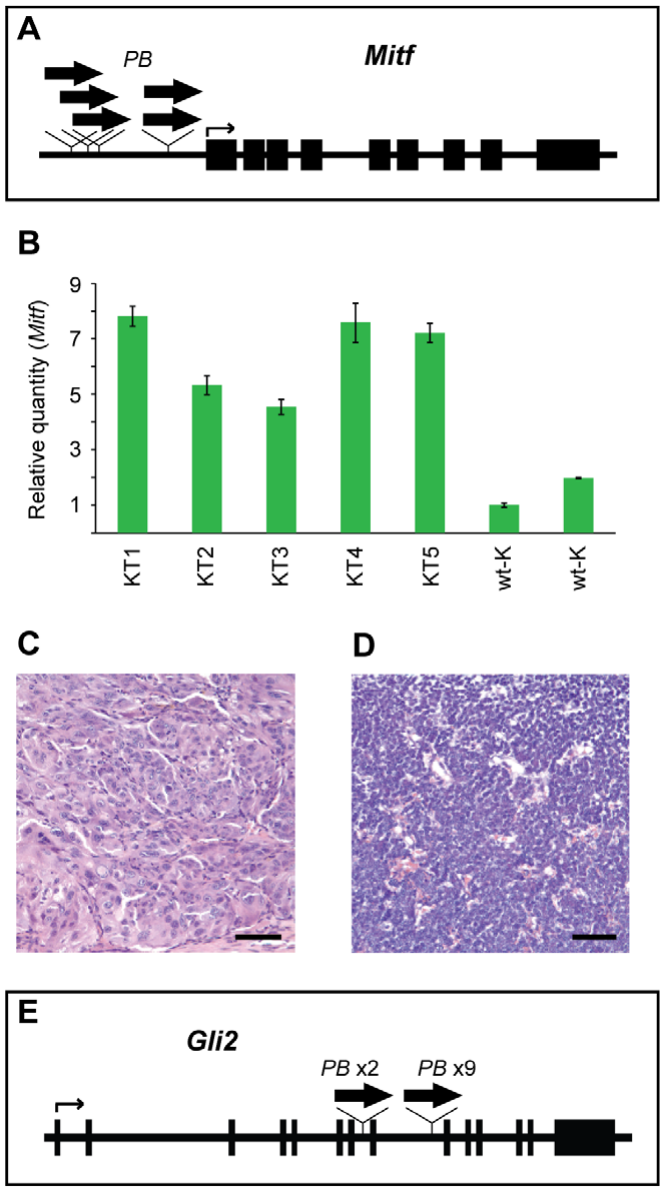
Identification of driver oncogenes in solid tumors from *Luc-PB[mut]7;Act-PBase* mice. (**A**) Insertions (wide arrows) upstream of the M-isoform of *Mitf* were mapped from five kidney tumors. (**B**) Quantitative PCR reveals that *Mitf* transcripts are upregulated in all five kidney tumors (KT1, KT2, KT3, KT4, and KT5) compared to two wild-type kidneys (wt-K). (**C**) Kidney tumors possessed similar histological profiles, featuring packets of spindle-shaped epitheliod cells, indicating carcinoma (scale bar, 50 µm). (**D**) Histological analysis shows SRBC morphology (scale bar, 100 µm). (**E**) Insertions (wide arrows) in intron 7 or 8 of *Gli2* were mapped from eleven SRBC tumors.

In five kidney tumors, we identified common insertions in *Mitf*. The mutator transposons in all five tumors were inserted in the coding direction, upstream of the translational start of the M-isoform, suggesting overexpression of *Mitf* in these tumors (Figure 2A). Indeed, quantitative PCR revealed that *Mitf* was upregulated in these kidney tumors as compared to two wild-type kidney samples (Figure 2B). Histopathology of the kidney tumors revealed an expansion of nests of pleomorphic spindle and epithelioid cells suggestive of carcinoma (Figure 2C).

Notably, two other members of the MiT family of basic-helix-loop-helix/leucine zipper transcription factors, *TFE3* and *TFEB*, have been recurrently affected by translocations that result in the upregulation of these transcription factors in human pediatric renal cell carcinoma [22]–[26]. Since TFE3, TFEB, and MITF are highly homologous and bind to the same DNA consensus sequence, it is likely that they can substitute for each other as oncogenes [27]. Together, these data suggest that *MITF* upregulation is an important driver for kidney tumor formation.

We also identified *Gli2*, a downstream effector of the *Hedgehog* (*Hh*) signaling pathway [28], as a common insertion site in SRBC tumors. We mapped coding-direction insertions in intron 7 or intron 8 from 11 tumors displaying SRBC morphology (Figures 2D and 2E). Gli2 contains a repressor domain in the amino-terminus, and expression of the C-terminal portion of Gli2 has previously been shown to result in the constitutive transcriptional activation of downstream target genes [29], [30]. In these sarcomas, it is likely that the *Gli2* insertions lead to the expression of constitutive transcriptional activator forms of Gli2 that drive tumor formation. In fact, constitutive Hedgehog signaling in Gorlin's syndrome due to mutation in *PTCH* leads to an increased propensity to develop an SRBC tumor, rhabdomyosarcoma [31], [32]. Furthermore, activation of Gli1 (a Gli2 transcriptional target) seems to be a major downstream effector of the EWS-FLI1 oncoprotein which drives another SRBC tumor, Ewing Sarcoma [33], [34]. Together these data strongly support the notion that constitutive Hedgehog signaling is a critical driver in SRBC tumor formation, with overexpression of truncated *Gli2* being one mechanism. Thus, the identification of cancer genes with known human relevance validates the utility of our low-copy mutator for uncovering disease genes.

### PB somatic mutagenesis with an activated RFP cell tracker

**Figure 3:**
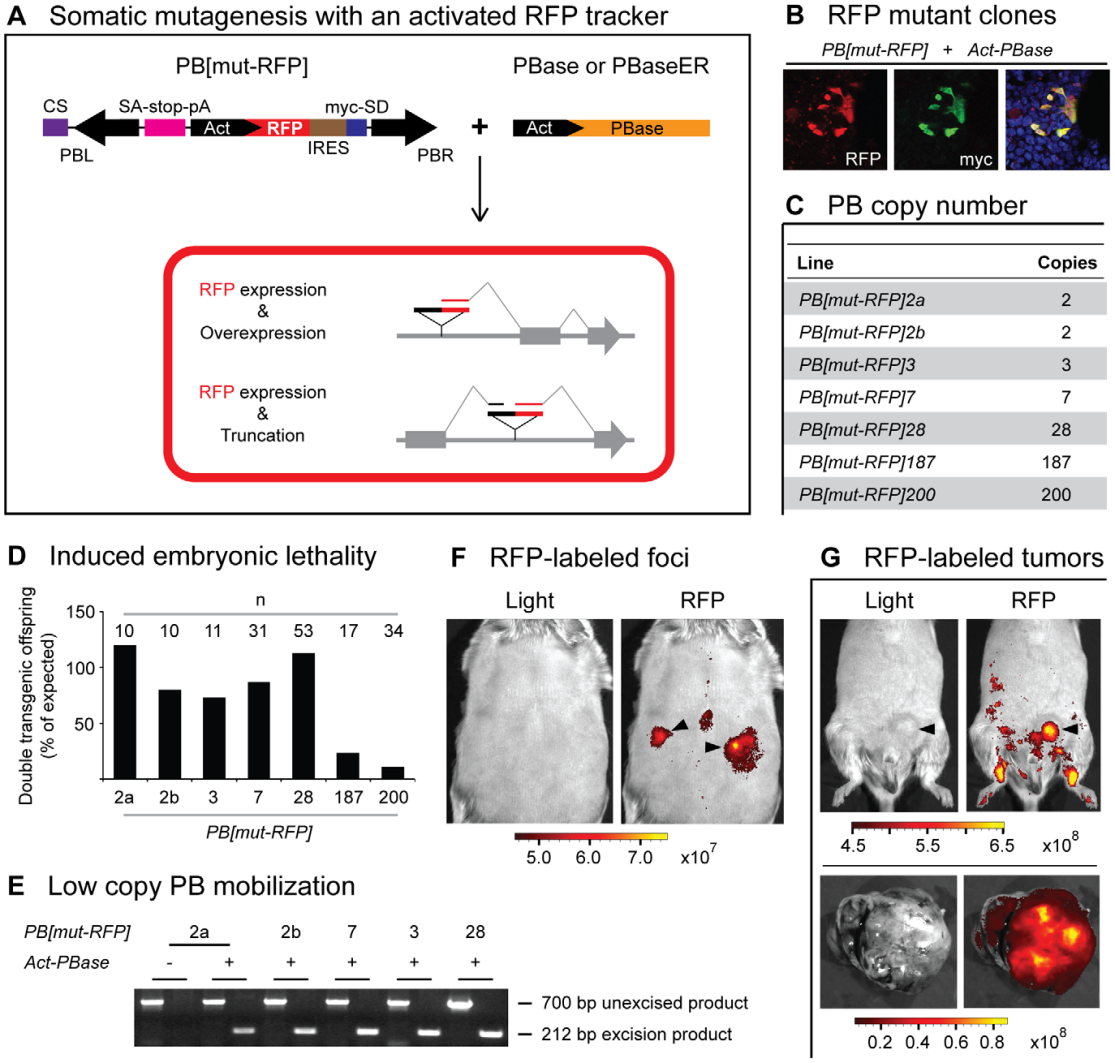
Simultaneously inducing mutations and tracking mutated cells with a PB transposon system. (**A**) *PB[mut-RFP]* couples RFP expression with ectopic expression of a downstream gene or partial gene transcript via the IRES (brown box). CS (purple box) prevents unwanted RFP expression from transgene concatamers. (**B**) *PB[mut-RFP]* mobilization in HEK 293 cells induces RFP-positive clones that colocalize with myc-tagged proteins ectopically expressed by the mutator transposon. (**C**) Transgenic lines carrying two to 200 copies of *PB[mut-RFP]* were generated. (**D**) Significant PB transposition-induced embryonic lethality was observed when high-copy lines were crossed to *Act-PBase*. (**E**) Low-copy *PB[mut-RFP]* lines crossed to *Act-PBase* mobilized the mutator transposon as determined by PCR with primers directed against sequences flanking the transposon. A 212-bp excision product was specifically detected in double transgenics but not in single transgenic littermates. A 700-bp unexcised product was observed in all *PB[mut-RFP]* mice. (**F**) Shown here in the dorsal skin of a *PB[mut-RFP]2a;Act-PBase* mouse, RFP-positive foci can be detected *ex vivo* over background signal. Units, photons·s^−1^·sr^−1^·ìM^−1^. (**G**) RFP-marked tumor formation (arrowhead) can be visualized *ex vivo* as seen in the upper panels showing a *PB[mut-RFP]2b;Act-PBase* mouse. In the lower panels, a stomach tumor dissected from a *PB[mut-RFP]7;Act-PBase* mouse is strongly RFP-positive. Units, photons·s^−1^·sr^−1^·µM^−1^.

In somatic mutagenesis, the ability to track mutated cells via the same transgenic line used to induce mutations opens up many experimental possibilities. We thus added new elements in our PB mutator transposon to label the mutant cells. The RFP coding sequence and an internal ribosome entry site (IRES) were inserted between the Actin promoter and myc-SD, which contains start codons in all three reading frames followed by a splice donor, of *PB[mut]* (Figure 3A, *PB[mut-RFP]*). Following insertions into introns or 5′ regulatory regions, production of bicistronic pre-mRNA containing RFP, IRES, the engineered myc-SD exon, and downstream endogenous intron and exon sequences can be initiated by the Actin promoter. After splicing of the myc-SD exon to downstream endogenous splice acceptors, the bicistronic transcripts enables the expression of RFP and the ectopic expression of endogenous proteins (Figure 3A). When we tested this mutator in cultured cells, the activated RFP marker was co-expressed in cells that ectopically expressed downstream endogenous proteins that incorporated the engineered myc-SD exon (Figure 3B). In order to ensure that *PB[mut-RFP]* does not express RFP from a transgenic concatamer, a concatamer silencer (CS) was inserted outside of the transposon (Figure 3A). The CS contains a splice acceptor followed by stop codons in all three reading frames to terminate transcripts initiated by the Actin promoter from transgene concatamers. To prevent alternative splicing–mediated exclusion of the CS splice acceptor, a sequence forming a pre-tRNA-like (ΔAC) structure was cloned after the stop codons. This substrate is efficiently recognized and cleaved by RNase P ensuring that pre-mRNA which contain the CS do not form mature transcripts [35].

We generated seven transgenic lines varying in transposon copy number from two copies to 200 copies (Figure 3C) and found that none were RFP-positive, indicating that the CS successfully prevents undesired RFP production. When the two highest copy lines were crossed to the *Act-PBase* line, live-born double transgenic progeny were rarely observed (Figure 3D), suggesting that the mutagenic transposons can be mobilized by *Act-PBase* during embryonic development and can induce significant deleterious effects and embryonic lethality. The mobilization of the low copy *PB[mut-RFP]* lines was also confirmed by excision PCR (Figure 3E). Importantly, mutagenesis using either of the two-copy or the seven-copy *PB[mut-RFP]* lines crossed to *Act-PBase* mice resulted in the appearance of RFP-positive patches (Figure 3F) and triggered tumorigenesis (Figure 3G), validating these three low copy *PB[mut-RFP]* lines for somatic mutagenesis with an activated cell tracker.

### Tissue-specific PB somatic mutagenesis screening with an activated reporter and tracker (PB-SMART screen)

**Figure 4:**
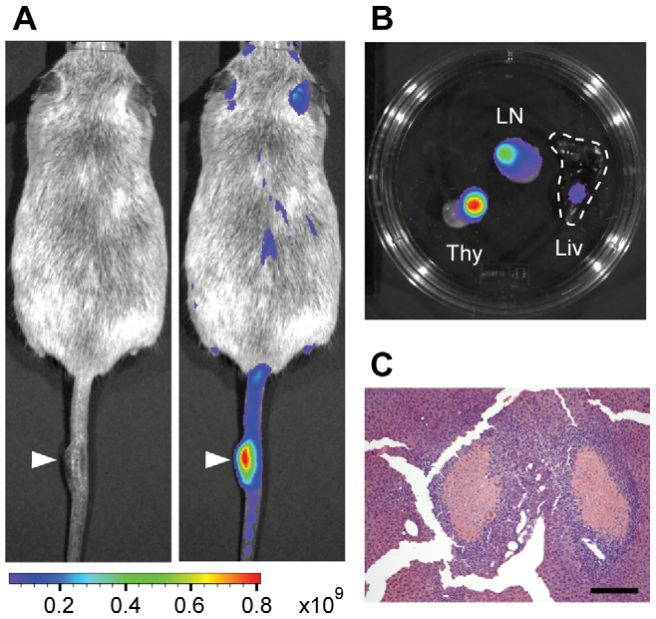
Tracking tumor formation and infiltration with *Luc-PB[mut]7*. (**A**) Luciferase signal labels a melanoma in this *Luc-PB[mut]7;Act-PBase;Braf^V600E^;Tyr-CreER* mouse (white arrowheads). The left panel shows a light image while the right panel overlays the luciferase signal. (**B**) Luciferase signal enabled the identification of tumor cell infiltration into distant organs of a *Luc-PB[mut]7;Act-PBase;Pten^+/-^* mouse that developed lymphoma. The enlarged thymus (Thy) and lymph nodes (LN) were strongly luciferase-positive. Strong signal was also seen emanating from a distinct portion of the liver (Liv, white dashed line). (**C**) H&E staining of liver from the luciferase-positive region reveals extensive lymphocyte infiltration (scale bar, 200 µm).

We have shown that the collective luciferase signal in tissues generated by *Luc-PB[mut]7* can act as a robust reporter of mutagenesis activity. Since *Luc-PB[mut]7* is also a mutator, the luciferase signal could also be used to mark the mutant cells. Given that *Luc-PB[mut]7* is a low copy line and not every cell in a tissue will be luciferase positive, we reasoned that clones or patches of mutant cells will have elevated signals and could be detected through *ex vivo* imaging. In this sense, the luciferase reporter also behaves as a tracker for mutant cells. By monitoring *ex vivo* luciferase signal, we identified clonal patches in the skin and tracked the appearance of melanomas in *Luc-PB[mut]7;Act-PBase;Braf^CA^;Tyr-CreER* mice (Figures 4A and 6A; also see below). In addition, the luciferase signal enabled the identification of tumor cell infiltration into distant organs of a *Luc-PB[mut]7;Act-PBase;Pten^+/-^* mouse that developed lymphoma (Figure 4B). The enlarged thymus and lymph nodes were strongly luciferase-positive due to the dense clustering of cells that activated the luciferase reporter (Figure 4B). Strong signal was also seen emanating from a distinct portion of the liver (Figure 4B). Histology revealed that the luminescent spot was indeed infiltration of lymphocytes (Figure 4C), and we mapped identical transposon insertions from the liver infiltration and other tumor sites, indicating that the cells originated from the same clone. Thus, the *Luc-PB[mut]7* line is a mutant clone tracker for monitoring clonal expansion, tumor formation, and infiltrations of cells into foreign tissues.

**Figure 5:**
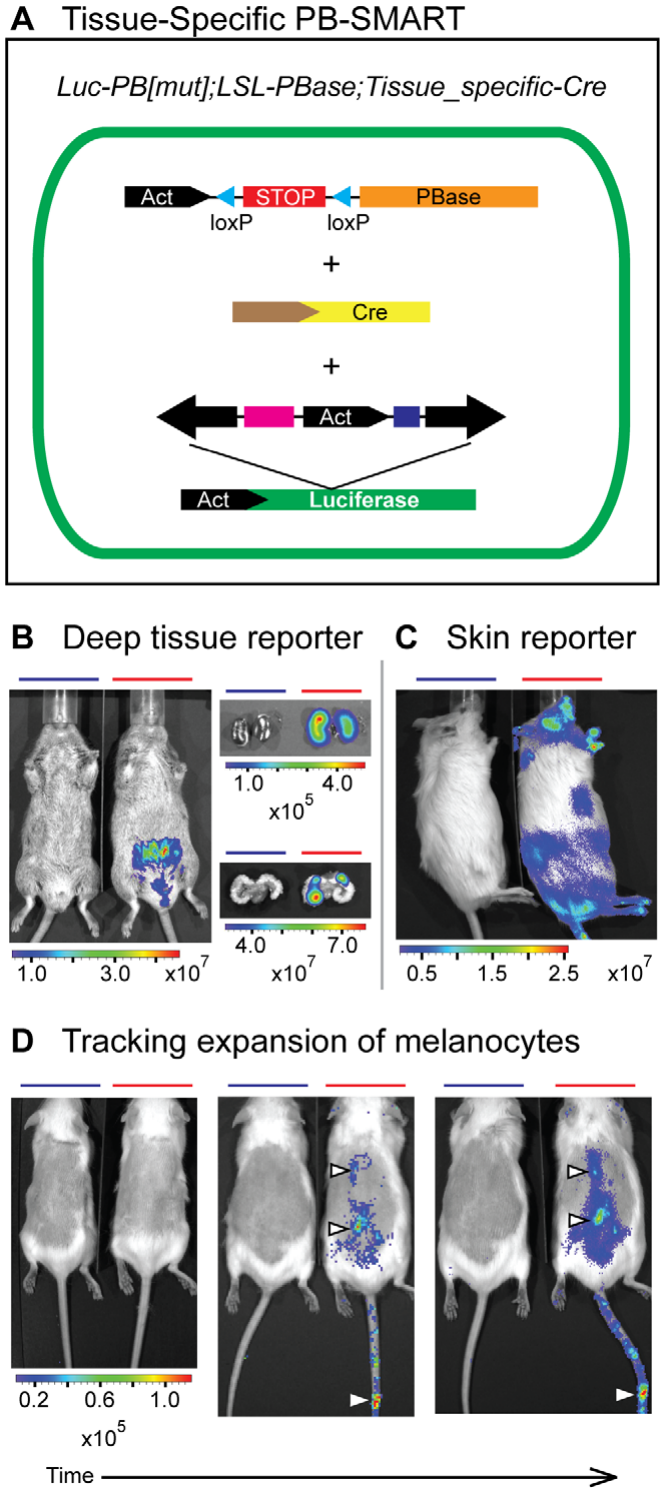
Tissue-specific PB-SMART. (**A**) Activation of the conditional *LSL-PBase* enables tissue-specific mutagenesis by crossing transgenic mice to available Cre lines. (**B**) *Luc-PB[mut]7;LSL-PBase;Ksp-Cre* transgenics (red bars) undergo PB mutagenesis specifically in the seminal vesicles and kidneys, reported by luciferase signal. Control mouse: *Luc-PB[mut]7;LSL-PBase* (blue bars). Units, photons·s^−1^·cm^−2^·sr^−1^. (**C**) PB mutagenesis throughout the skin of *Luc-PB[mut]7;LSL-PBase;K14-Cre* mice (red bar) is reported by luciferase signal. Control mouse: *Luc-PB[mut]7;K14-Cre* (blue bar). Units, photons·s^−1^·cm^−2^·sr^−1^. (**D**) *Tyr-CreER* conditionally activates *Braf^V600E^* and PB mutagenesis specifically in melanocytes of quadruple transgenic mice (*Luc-PB[mut]7;LSL-PBase;Braf^CA^;Tyr-CreER*, red bars). The expansion of this cell population at three, nine, and eleven months (left, middle and right, respectively) is tracked *ex vivo* by luciferase signal. Control mouse: *Luc-PB[mut]7;BrafCA;Tyr-CreER* (blue bars). Units, photons·s^−1^·cm^−2^·sr^−1^.

We further expanded the PB-SMART system for tissue-specific genetic screens. To allow the system to be readily adopted for screens in many types of tissues and cells, we generated transgenic mice in which *PBase* or *PBaseER* can be activated by tissue-specific Cre by inserting a Stop-pA sequence flanked by loxP sites between the Actin promoter and the transposase coding sequence (Figure 5A, *LSL-PBase, LSL-PBaseER*). When these double transgenics were crossed into lines expressing Cre in the kidney and genitourinary tract or the skin epithelium [36], [37], correct targeting of mutagenesis to these tissues was confirmed by the luciferase reporter (Figures 5B and 5C).

To ask whether *Luc-PB[mut]7* can be used to track mutation-driven expansion of cells, we utilized the *Tyr-CreER* line, which expresses CreER specifically in melanocytes [38], one of the least densely clustered cell types in the body. *Tyr-CreER* has been used to conditionally activate *Braf^V600E^*, thereby promoting melanocyte proliferation [20], [21]. We generated *Luc-PB[mut]7;LSL-PBase;Braf^CA^;Tyr-CreER* quadruple transgenic mice and assayed whether luciferase can track mutation-driven expansion of cells *ex vivo*. After administering tamoxifen, we were able to detect luciferase signal in melanocytes in the ears, tail, and dorsal trunk and also track the expansion of the positive cells over time (Figure 5D).

**Figure 6:**
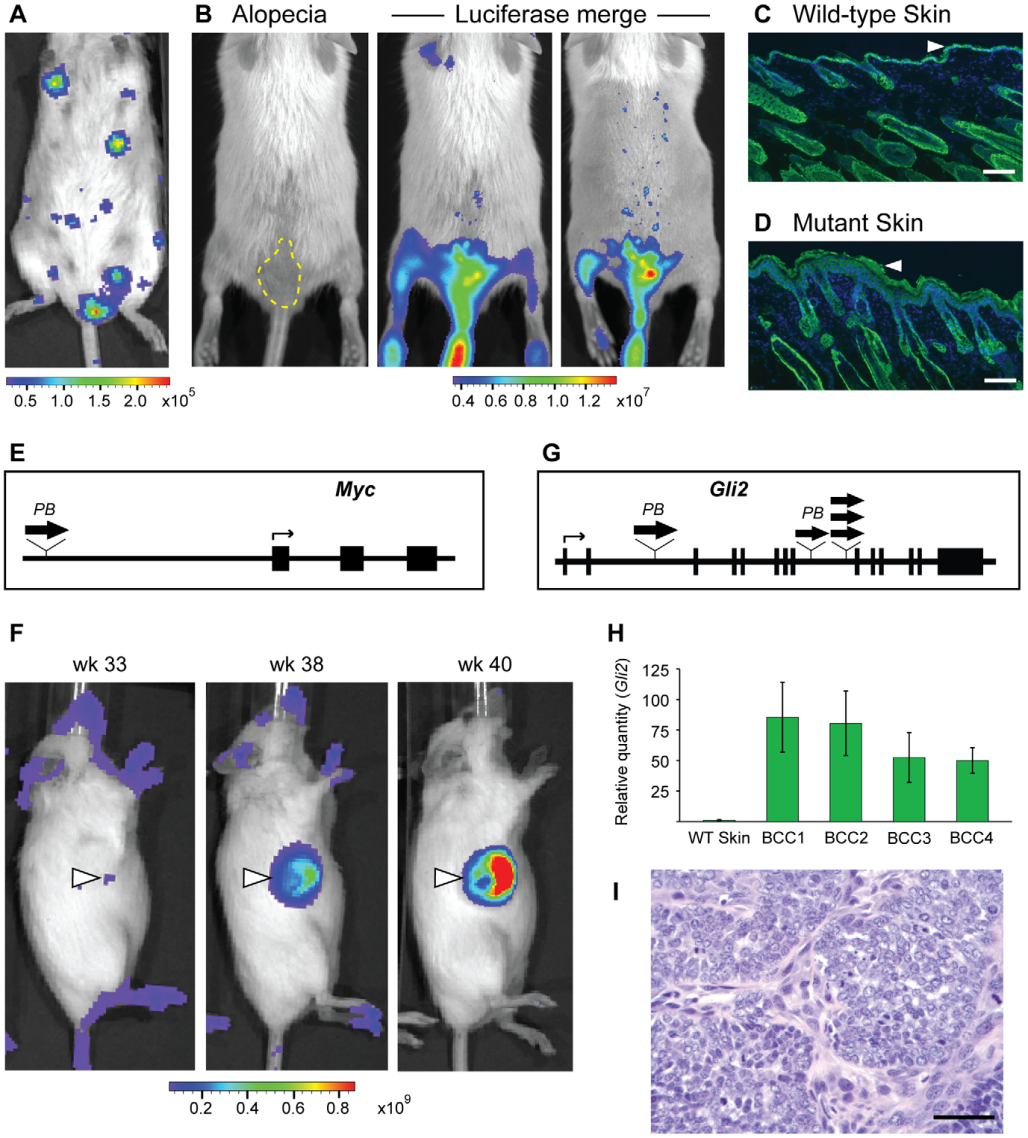
Tissue-specific induction of skin phenotypes by Cre-activated PB mutagenesis. (**A**) Luciferase signal marks clones of mutated cells in *Luc-PB[mut]7;LSL-PBase;K14-Cre* mice. (**B**) PB-induced alopecia in a *PB[mut]7;LSL-PBase;K14-Cre; Pten^lox/+^* mouse, (yellow dashed line) shows strong luciferase signal corresponding to hair loss (middle). Fully shaved, luciferase signal demarcates the specific region of alopecia (right). (**C**) Cytokeratin staining (green) of wild-type dorsal skin shows the normal thickness of the mouse epidermis (white arrowhead). DAPI (blue) marks cell nuclei. (**D**) Staining as in (c) from the region of alopecia reveals substantial thickening of the epidermis (white arrowhead). (**E**) A transposon insertion upstream of *Myc* was mapped to the affected region of skin. (**F**) Skin-specific mutagenesis in *Luc-PB[mut]7;LSL-PBase;K14-Cre* mice induces tumor formation and luciferase-labeled tumor cells. The white arrow corresponds to region of luciferase signal that develops into a larger tumor over time. Units, photons·s^−1^·cm^−2^·sr^−1^. (**G**) Insertion sites (wide arrows) in *Gli2* were mapped in multiple skin tumors. (**H**) By quantitative PCR, *Gli2* transcripts were upregulated in each skin tumor (BCC1, BCC2, BCC3, and BCC4) compared to WT skin. (**I**) The skin tumors bear resemblance to human basal cell carcinoma according to histological analysis, consistent with the activation of the *Hedgehog* pathway (scale bar, 50 µm).

To further illustrate that *Luc-PB[mut]7* can be used to track clonal expansion in specific cell types, we targeted the PB-SMART system to the mouse skin. We generated *Luc-PB[mut]7;LSL-PBase;K14-Cre* mice which express Cre specifically in the basal keratinocytes of the epidermis. This compartment contains the stem and progenitor cells that give rise to the hair follicle, sebaceous gland, and interfollicular epidermis [39]. We reasoned that if a keratinocyte stem or progenitor cell were to be targeted by PB-SMART, over time an entire clonal unit would become luciferase positive. Indeed, the appearance of distinct luciferase clusters could be detected in these mice (Figure 6A).

Skin-specific PB-SMART resulted in tumor development as well as other morphological phenotypes. For example, we observed hair loss on dorsal skin, resulting from PB mutagenesis and marked by luciferase signal (Figure 6B). Keratin staining of frozen sections revealed a striking epidermal thickening in the affected region (Figures 6C and 6D). We mapped a transposon insertion upstream of *Myc* in the affected skin (Figure 6E). This is consistent with previous reports that in *K14-c-Myc* transgenic mice, epidermal stem cell proliferation and differentiation results in hair loss, hyperproliferation, and thickening of the interfollicular epidermis [40], [41].

We further analyzed skin tumors that were induced and labeled by skin-specific PB-SMART. We were able to observe tumors in *Luc-PB[mut]7;LSL-PBase;K14-Cre* mice starting at four months of age. Using the e*x vivo* reporter system, we can detect regions of luciferase activity prior to tumor formation, that correspond to the later development of tumors (Figure 6F). In four of these tumors, we identified transposon insertions in *Gli2* (Figure 6G). Quantitative PCR confirmed that *Gli2* mRNA levels were increased in these tumors as compared to wild type skin (Figure 6H). Constitutive activation of the *Hh* pathway is characteristically seen in basal cell carcinoma (BCC) [42], which arises from stem cells in the epidermis and hair follicle [43], [44]. Furthermore, histopathology of the skin tumors revealed palisading basaloid cells forming tumor nests and retraction of the stroma from the tumor islands in formalin-fixed sections similar to human BCC (Figure 6I). Together, these data show that the PB-SMART system can be deployed to conduct tissue-specific genetic screens and identify causative mutations relevant to human disease.

## Discussion

In the data presented, we have taken advantage of the PB transposon's properties to create a somatic mutagenesis system, PB-SMART, to simultaneously mutate and mark cells for conducting tissue-specific genetic screens in mice. In somatic screens for cancer genes, PB-SMART offers advantages over other TIM systems. Our system allows one to detect tumors before their morphological manifestation and to monitor tumor growth and dissemination *ex vivo*. Importantly, it allows one to detect tumor formation *ex vivo* such that only tumor-positive animals will be sacrificed in a screen. This is particularly critical for screens of internal cancers as previous screens required sacrificing large numbers of experimental animals at arbitrary time points to search for tumors. The visible cell markers also enable mutant clones in tissues to be readily identified and continuously monitored over time. This is essential for the detection of phenotypes other than tumors and provides many experimental advantages for visualizing proliferation defects, tracking cell migration, tracing cell lineages, and analyzing other clonal behavior *in vivo*. As proof-of-principle examples, we demonstrated that the system can be used to identify phenotypes in PB mutant clones, detect cell infiltration, and track the expansion of rare cell populations. In addition to these examples, the luciferase and RFP mutator lines can be utilized in other ways to confer specific experimental advantages according to the biological process to be surveyed. For example, luciferase is highly useful for detection and tracking of solid tumors originating from deep tissues. On the other hand, RFP enables detection and isolation of cells via fluorescence-activated cell sorting which would be particularly useful for capturing disseminated tumor cells in the bloodstream. As an additional resource to facilitate use by the research community, we will provide further detailed characterization of these and other recently developed PB-SMART lines on our lab website (http://info.med.yale.edu/genetics/xu).

The luciferase reporter integrated into the PB-SMART system also allows mutagenesis efficiency to be visually assessed at the onset of a genetic screen without sacrificing animals. Furthermore, we have found that luciferase signal activated by PB mutagenesis is highly indicative for the successful outcome of a mutagenesis screen, providing a valuable predictive tool. Genetic screens in mice are a significant investment. Since phenotypes in many screens take significant time to manifest, it is a tremendous advantage to have a predictive indicator at the beginning of the experiments.

PB-SMART is also a highly efficient mutagenesis system. Utilizing PB's large payload capacity, we engineered elements in PB to ectopically express genes or parts of genes and to terminate transcripts upon insertion in many genomic contexts, thereby creating highly mutagenic transposons. Together with PB's high transposition efficiency and genome-wide distribution, PB-SMART is able to induce somatic phenotypes with low copy (seven or fewer) transposons. The efficiency of the system is highlighted by the fact that a broad spectrum of tumors is induced with only seven copies of transposon. Importantly, low transposon copy numbers allow for more straightforward identification of causative insertions because there are fewer bystander insertions. We found that over 64% of insertions in tumors are mapped in introns, exons, or within 10 kb of genes. This is a remarkably high gene insertional frequency indicative that there is a selective advantage for PB gene insertions in tumors. In two tumor types that have not previously been found in somatic mutagenesis screens, we used common insertion site analysis to identify *Mitf* as a driver in kidney tumor formation and truncated *Gli2* as a driver of SRBC tumors. Thus, PB-SMART is a powerful tool to identify mutations relevant to human disease.

For the creation of a tissue-specific mutagenesis system that can be readily used by the research community for a variety of tissue and cell types, we generated conditional transposase transgenic lines that can be activated by Cre (*LSL-PBase* and *LSL-PBaseER*). We showed that mutagenesis can be targeted specifically to the skin or internal organs by crossing to tissue-specific Cre lines. Temporal control of tissue-specific mutagenesis can also be achieved by using *LSL-PBaseER* or by crossing to available CreER lines, as we demonstrated in melanocytes with *Tyr-CreER*. Given that specific Cre or CreER lines have been generated for almost every mouse tissue being studied, our system will make it possible to target any tissue for genetic screens. The combination of conditional transposase lines and low copy PB mutator lines could be used to target the germline for the generation of stable and heritable mutations for traditional screens. More importantly, mutations targeted at the germline could also be used to conduct F1 screens for developmental and physiological phenotypes including embryonic and adult defects.

In summary, the ability to induce somatic mutations, report mutagenesis levels, and track mutated cells permits many types of applications and analyses that are not possible with current TIM systems. Because induction and labeling of mutant cells are simultaneously achieved by the same mutator transposon, the design not only links the two events, but also simplifies genetics by minimizing the number of transgenes involved. Furthermore, PB-SMART also integrates the conditional transposase, which enables TIM screens to be conducted for any tissue in the mouse by a single genetic cross to a tissue-specific Cre line. The ability to screen thousands of clones in a single animal in only one generation makes it possible to achieve genome-wide interrogation with a small number of animals in a short period of time. The development of PB-SMART thus empowers individual investigators to decipher the genetic basis of biology and disease by forward genetic screens.

## Methods

### Ethics Statement

All experiments were approved by and conducted in compliance with the Yale Animal Resources Center and the Institutional Animal Care and Use Committee under protocol number 2008-10230.

### Plasmid construction

The *Luc-PB[mut]7* and *PB[mut-RFP]* transposons were generated from elements in the plasmids *PB[Act-RFP]* [3], *pIRES* (Clontech), *pBS302* (Life Technologies), *pΔAC* [35], and *pGL3* (Promega). Myc-SD was PCR amplified from an oligonucleotide containing ATG transcriptional initiation codons, myc epitope sequences in all three reading frames, and a splice donor sequence. CS and part of SA-stop-pA were PCR amplified from an oligonucleotide containing a splice acceptor and stop codons in all three reading frames (sequences available upon request). For *PB[mut-RFP]*, loxP sites were included to flank the mutagenic *Act-RFP-IRES-myc-SD* cassette. All elements were constructed on a *pBlueScript II SK+* (Stratagene) backbone before cloning between PBL and PBR transposon arms to yield the final plasmid. For *Luc-PB[mut]*, luciferase from *pGL3* was introduced outside PBL and PBR by overlapping PCR followed by cloning into the XhoI and NotI sites in *PB[Act-RFP]*.

*LSL-PBase* plasmid was generated from DNA elements in the plasmids *Act-PBase* [3] and *pBS302*. A 1.5-kb transcriptional termination sequence flanked by loxP sites was obtained from the *pBS302* plasmid by digestion with EcoRI and SpeI, and subsequently cloned between the Actin promoter and PBase coding sequence in the *Act-PBase* plasmid. Two copies of the chicken *β-globin* HS4 core enhancer from the plasmid *pNI-CD* were also cloned upstream of the Actin promoter.

*PBaseER* was generated by inserting a 12-nucleotide linker sequence that encodes a four-amino acid, positively charged linker (5′-AGGCGCGCCCCC-3′) between the *PBase* coding sequence and the mutated tamoxifen-responsive mouse estrogen receptor hormone-binding domain coding sequence [45]. *PBaseER* was subsequently cloned after the Actin or LSL promoter.

### Generation of transgenic mice

*Luc-PB[mut]* and *PB[mut-RFP]* plasmids were linearized by NotI or PspXI. *LSL-PBase* plasmid was linearized by SalI and StuI. *Act-PBaseER* was linearized by SalI and BamHI. After gel purification (Qiagen), linear DNA was injected into C57BL/6J x SJL/J F2 hybrid one-cell embryos. Founders were screened for the presence or absence of the transgene by PCR analysis (see Table S1 for primer sequences).

### Mouse lines, breeding and husbandry

*Act-PBase, Tyr-CreER, Braf^CA^, Pten^lox/+^, Ksp1.3-Cre (Tg(Cdh16-cre)91Igr/J)*, and *K14-Cre (Tg(KRT14-cre)1Amc/J)* lines have been reported previously [3], [20], [36], [37]. *Pten^+/^* were generated by mating a *Pten^lox/+^* male to a *Pbsn-Cre4 (B6.Cg-Tg(Pbsn-cre)4Prb)* female to induce recombination in the germline [46]. Transgenic mice were backcrossed to the FVB/NJ mouse strain. Animals were fed a standard rodent diet.

### Administration of tamoxifen to mice

100 mg/ml tamoxifen was dissolved in ethanol and diluted 1∶10 in sunflower seed oil (for intraperitoneal injection) or 1∶100 in water (for tamoxifen-water bottles). Newly weaned mice were injected with 100 µl (1 mg) tamoxifen-in-oil mixture once per day for five consecutive days. For tamoxifen-water bottles, mice were constantly kept on 100 µg/ml tamoxifen-water.

### Histology and immunofluorescence

For paraffin-embedded sections, tissues were dissected from euthanized mice and fixed in 10% neutral-buffered formalin overnight, dehydrated in a series of increasing concentrations of ethanol and xylenes, embedded in paraffin, sectioned, and stained with hematoxylin and eosin. For frozen sections, dissected tissues were OCT-embedded, frozen, sectioned, and fixed in 4% paraformaldehyde (PFA). For immunofluorescence staining of cytokeratin, OCT sections were fixed for 10 min in 4% PFA in PBS and washed three times for 5 min in PBS. Sections were permeabilized with 0.1% Triton for 15 min, washed three times for 5 min in PBS, and blocked for one hour with blocking solution (5% NGS, 1% BSA, and 0.1% Triton X-100 in PBS). Rabbit anti-cow cytokeratin wide spectrum screening antibody (1∶250, Dako) was added overnight at 4°C. Alexa Fluor 488 goat anti-rabbit IgG (1∶2000; Invitrogen) was used for detection of primary antibodies and nuclei were stained with DAPI. Blocking solution and antibody incubation steps were performed in a humidified chamber.

### *Ex vivo* mouse imaging

Mice were sedated using an isoflurane vaporizer. For *ex vivo* visualization of RFP, sedated *PB[mut-RFP]* control and *PB[mut-RFP];Act-PBase* mice were shaved and immediately imaged side-by-side in the IVIS Spectrum (Xenogen) by epifluorescence. Autofluorescence in shaved regions was quantitated in control mice to adjust epifluorescence imaging parameters. To visualize *in vivo* luciferase activity using the IVIS Spectrum, sedated mice were weighed, treated with 150 mg luciferin per kg body weight via intraperitoneal injection, and subsequently imaged 15 minutes after luciferin injection.. Heart rate and respiration were closely monitored and body temperature was regulated throughout the entire procedure.

### Linker-mediated PCR identification of PB insertion sites

LM-PCR mapping of PB mutator insertion sites was performed using a previously developed protocol [47] with the following modifications. Two µg of mouse genomic DNA were digested overnight with Sau3AI or MspI and subsequently heat inactivated. Double-stranded Sau3AI-specific or MspI-specific linkers were ligated to digested genomic DNA in an overnight reaction at 18°C. Ligation products were digested in a second reaction by MfeI to eliminate adaptor-ligated DNA fragments containing mutator transposon at its original site of insertion. Following heat inactivation of enzyme and purification of digestion products (Qiagen), a primary PCR reaction, followed by a nested PCR reaction, were performed. Amplification products were purified (Qiagen), TA-cloned into the *pGEM-T-Easy* vector (Promega), and transformed in competent cells. Colonies were screened for inserts by colony PCR using the T7 and SP6 primers and those containing inserts sequenced by traditional Sanger sequencing. Oligonucleotides used in this procedure can be found in Table S1.

### Sequence analysis and annotation

Insertion sites were mapped by Blatting against the mouse genome with UCSC Genome Bioinformatics Site (http://genome.ucsc.edu/).

### RNA isolation and quantitative PCR

RNA was isolated from tumors or wild type tissues (Qiagen). 1–2 µg of RNA was then used with oligo-dT primer and Superscript III (Invitrogen) to synthesize cDNA. Quantitative PCR with SYBR green in the StepOne Real-Time PCR instrument (Applied Biosystems) was used to determine transcript levels with the relative standard curve method. Primers are detailed in Table S1.

### Excision PCR

Quantitative PCR was used to determine the relative excision levels of *Luc-PB[mut]* from spleen genomic DNA. Mobilization of *PB[mut-RFP]* from the concatamer was determined by traditional PCR using primers designed against the plasmid sequences flanking the PB[mut-RFP] transposon. Primer sequences are detailed in Table S1.

### Cell culture

HEK 293 cells were grown in DMEM containing 10% FBS under standard conditions. Cells were transfected with circular plasmids using Lipofectamine 2000 according to the manufacturer's instructions (Invitrogen). To select neomycin-resistant colonies, Geneticin (G418) was added to the culture medium at a concentration of 500 µg/ml. For two weeks, culture medium was replaced every 3–4 days and G418 was resupplemented each time. To induce PBaseER activity, 4-hydroxtamoxifen (4-OHT) was added to culture medium to obtain the desired final concentration.

### Confocal microscopy

HEK293 cells were seeded onto sterilized cover slips, cultured until 90% confluent, and then fixed in 4% paraformaldehyde (PFA) for 15 minutes at room temperature. Following permeabilization using 0.1% Triton X-100 in PBS (PBST), cells were blocked with 5% normal goat serum at room temperature for 30 minutes and then incubated with primary antibodies. The ERα primary antibody (sc-542, Santa Cruz Biotechnology) and mouse monoclonal anti-myc 9E10 primary antibodies were used. FITC-conjugated secondary antibodies were used to visualize proteins. Samples were mounted in Vectashield containing DAPI (Vector Laboratories). Immunofluorescence images were collected using a Zeiss LSM Meta confocal microscope and processed using Adobe Photoshop.

### Methylene blue staining

To stain colonies in tissue culture dishes, culture medium was aspirated by vacuum and washed once in PBS. Subsequently, cells were fixed in ice cold methanol for 20 minutes at room temperature. Staining was performed using 0.2% methylene blue solution for one hour at room temperature. Following this step, the culture dishes were rinsed with distilled water to remove residual stains.

### Western blotting

Cells were lysed in lysis buffer and protein concentrations of lysates were determined by the BCA Assay (Pierce). Approximately 15 µg of total protein was loaded on a 10% SDS-PAGE gel. Protein was transferred onto the Trans-Blot Transfer Medium (Biorad). Nitrocellulose membranes were blocked at room temperature for 1 hour with 5% milk in PBS, then washed 3 times for 10 minutes in PBS and incubated with ERα primary antibody (sc-542, Santa Cruz Biotechnology) in PBS and 3% BSA overnight at 4°C. Membranes were washed 3 times for 10 minutes in PBS, blocked at room temperature for 1 hour with 5% milk in PBS, and incubated in HRP-conjugated secondary antibodies. Immunoreactive bands were visualized using Western Lightning *Plus*-ECL (Perkin-Elmer).

## Supporting Information

Figure S1 **Tamoxifen-dependent translocation of PBaseERto the nucleus drives PB transposition in HEK 293 cells.** (**A**) *Act-PBaseER* in transfected HEK 293 cells was detected by Western blotting using a polyclonal ERα antibody and displayed the expected size. (**B**) Immunofluorescent staining with ERα antibody revealed that PBaseER translocates to the nucleus after 24-hour 4-hydroxytamoxifen (4-OHT) treatment. (**C**) HEK 293 cells co-transfected with circular plasmids carrying PB with a neomycin resistance cassette (*PB[SV40-neo]*) and *Act-PBase* form G418-resistant clones after drug selection for two weeks, indicative of PBase-mediated transposition activity. (**D**) 4-OHT treatment is required for PBaseER-mediated transposon insertion. Compared to *Act-PBase*, *Act-PBaseER* mediates transposition at a lower efficiency.
(TIF)

Table S1 Primers used in this work for genotyping transgenic lines, analyzing PB excision, analyzing PB copy number, analyzing relative transcript levels, and mapping PB insertion sites.
(DOC)

## References

[1] Cooley L, Kelley R, Spradling A (1988). Insertional mutagenesis of the Drosophila genome with single P elements.. Science.

[2] Kleckner N, Roth J, Botstein D (1977). Genetic engineering in vivo using translocatable drug-resistance elements. New methods in bacterial genetics.. J Mol Biol.

[3] Ding S, Wu X, Li G, Han M, Zhuang Y (2005). Efficient transposition of the piggyBac (PB) transposon in mammalian cells and mice.. Cell.

[4] Ivics Z, Hackett PB, Plasterk RH, Izsvak Z (1997). Molecular reconstruction of Sleeping Beauty, a Tc1-like transposon from fish, and its transposition in human cells.. Cell.

[5] Wu S, Ying G, Wu Q, Capecchi MR (2007). Toward simpler and faster genome-wide mutagenesis in mice.. Nat Genet.

[6] Bender AM, Collier LS, Rodriguez FJ, Tieu C, Larson JD Sleeping beauty-mediated somatic mutagenesis implicates CSF1 in the formation of high-grade astrocytomas.. Cancer Res.

[7] Collier LS, Adams DJ, Hackett CS, Bendzick LE, Akagi K (2009). Whole-body sleeping beauty mutagenesis can cause penetrant leukemia/lymphoma and rare high-grade glioma without associated embryonic lethality.. Cancer Res.

[8] Collier LS, Carlson CM, Ravimohan S, Dupuy AJ, Largaespada DA (2005). Cancer gene discovery in solid tumours using transposon-based somatic mutagenesis in the mouse.. Nature.

[9] Dupuy AJ, Akagi K, Largaespada DA, Copeland NG, Jenkins NA (2005). Mammalian mutagenesis using a highly mobile somatic Sleeping Beauty transposon system.. Nature.

[10] Dupuy AJ, Rogers LM, Kim J, Nannapaneni K, Starr TK (2009). A modified sleeping beauty transposon system that can be used to model a wide variety of human cancers in mice.. Cancer Res.

[11] Keng VW, Villanueva A, Chiang DY, Dupuy AJ, Ryan BJ (2009). A conditional transposon-based insertional mutagenesis screen for genes associated with mouse hepatocellular carcinoma.. Nat Biotechnol.

[12] Rad R, Rad L, Wang W, Cadinanos J, Vassiliou G (2011). PiggyBac Transposon Mutagenesis: A Tool for Cancer Gene Discovery in Mice.. Science.

[13] Starr TK, Allaei R, Silverstein KA, Staggs RA, Sarver AL (2009). A transposon-based genetic screen in mice identifies genes altered in colorectal cancer.. Science.

[14] Lee T, Luo L (2001). Mosaic analysis with a repressible cell marker (MARCM) for Drosophila neural development.. Trends Neurosci.

[15] Pagliarini RA, Xu T (2003). A genetic screen in Drosophila for metastatic behavior.. Science.

[16] White RM, Sessa A, Burke C, Bowman T, LeBlanc J (2008). Transparent adult zebrafish as a tool for in vivo transplantation analysis.. Cell Stem Cell.

[17] Xu T, Rubin GM (1993). Analysis of genetic mosaics in developing and adult Drosophila tissues.. Development.

[18] Elick TA, Bauser CA, Fraser MJ (1996). Excision of the piggyBac transposable element in vitro is a precise event that is enhanced by the expression of its encoded transposase.. Genetica.

[19] de Wet JR, Wood KV, DeLuca M, Helinski DR, Subramani S (1987). Firefly luciferase gene: structure and expression in mammalian cells.. Mol Cell Biol.

[20] Dankort D, Curley DP, Cartlidge RA, Nelson B, Karnezis AN (2009). Braf(V600E) cooperates with Pten loss to induce metastatic melanoma.. Nat Genet.

[21] Dankort D, Filenova E, Collado M, Serrano M, Jones K (2007). A new mouse model to explore the initiation, progression, and therapy of BRAFV600E-induced lung tumors.. Genes Dev.

[22] Davis IJ, Hsi BL, Arroyo JD, Vargas SO, Yeh YA (2003). Cloning of an Alpha-TFEB fusion in renal tumors harboring the t(6;11)(p21;q13) chromosome translocation.. Proc Natl Acad Sci U S A.

[23] Kuiper RP, Schepens M, Thijssen J, van Asseldonk M, van den Berg E (2003). Upregulation of the transcription factor TFEB in t(6;11)(p21;q13)-positive renal cell carcinomas due to promoter substitution.. Hum Mol Genet.

[24] Sidhar SK, Clark J, Gill S, Hamoudi R, Crew AJ (1996). The t(X;1)(p11.2;q21.2) translocation in papillary renal cell carcinoma fuses a novel gene PRCC to the TFE3 transcription factor gene.. Hum Mol Genet.

[25] Weterman MA, Wilbrink M, Geurts van Kessel A (1996). Fusion of the transcription factor TFE3 gene to a novel gene, PRCC, in t(X;1)(p11;q21)-positive papillary renal cell carcinomas.. Proc Natl Acad Sci U S A.

[26] Argani P, Antonescu CR, Illei PB, Lui MY, Timmons CF (2001). Primary renal neoplasms with the ASPL-TFE3 gene fusion of alveolar soft part sarcoma: a distinctive tumor entity previously included among renal cell carcinomas of children and adolescents.. Am J Pathol.

[27] Haq R, Fisher DE Biology and clinical relevance of the micropthalmia family of transcription factors in human cancer.. J Clin Oncol.

[28] Grachtchouk M, Mo R, Yu S, Zhang X, Sasaki H (2000). Basal cell carcinomas in mice overexpressing Gli2 in skin.. Nat Genet.

[29] Sasaki H, Nishizaki Y, Hui C, Nakafuku M, Kondoh H (1999). Regulation of Gli2 and Gli3 activities by an amino-terminal repression domain: implication of Gli2 and Gli3 as primary mediators of Shh signaling.. Development.

[30] Roessler E, Ermilov AN, Grange DK, Wang A, Grachtchouk M (2005). A previously unidentified amino-terminal domain regulates transcriptional activity of wild-type and disease-associated human GLI2.. Hum Mol Genet.

[31] Gorlin RJ (1987). Nevoid basal-cell carcinoma syndrome.. Medicine (Baltimore).

[32] Johnson RL, Rothman AL, Xie J, Goodrich LV, Bare JW (1996). Human homolog of patched, a candidate gene for the basal cell nevus syndrome.. Science.

[33] Beauchamp E, Bulut G, Abaan O, Chen K, Merchant A (2009). GLI1 is a direct transcriptional target of EWS-FLI1 oncoprotein.. J Biol Chem.

[34] Joo J, Christensen L, Warner K, States L, Kang HG (2009). GLI1 is a central mediator of EWS/FLI1 signaling in Ewing tumors.. PLoS One.

[35] Yuan Y, Altman S (1995). Substrate recognition by human RNase P: identification of small, model substrates for the enzyme.. EMBO J.

[36] Dassule HR, Lewis P, Bei M, Maas R, McMahon AP (2000). Sonic hedgehog regulates growth and morphogenesis of the tooth.. Development.

[37] Shao X, Somlo S, Igarashi P (2002). Epithelial-specific Cre/lox recombination in the developing kidney and genitourinary tract.. J Am Soc Nephrol.

[38] Bosenberg M, Muthusamy V, Curley DP, Wang Z, Hobbs C (2006). Characterization of melanocyte-specific inducible Cre recombinase transgenic mice.. Genesis.

[39] Lechler T, Fuchs E (2005). Asymmetric cell divisions promote stratification and differentiation of mammalian skin.. Nature.

[40] Arnold I, Watt FM (2001). c-Myc activation in transgenic mouse epidermis results in mobilization of stem cells and differentiation of their progeny.. Curr Biol.

[41] Waikel RL, Kawachi Y, Waikel PA, Wang XJ, Roop DR (2001). Deregulated expression of c-Myc depletes epidermal stem cells.. Nat Genet.

[42] Epstein EH (2008). Basal cell carcinomas: attack of the hedgehog.. Nat Rev Cancer.

[43] Wang GY, Wang J, Mancianti EH Basal cell carcinomas arise from hair follicle stem cells in Ptch1(+/-) mice.. Cancer Cell.

[44] Youssef KK, Van Keymeulen A, Lapouge G, Beck B, Michaux C Identification of the cell lineage at the origin of basal cell carcinoma.. Nat Cell Biol.

[45] Littlewood TD, Hancock DC, Danielian PS, Parker MG, Evan GI (1995). A modified oestrogen receptor ligand-binding domain as an improved switch for the regulation of heterologous proteins.. Nucleic Acids Res.

[46] Wu X, Wu J, Huang J, Powell WC, Zhang J (2001). Generation of a prostate epithelial cell-specific Cre transgenic mouse model for tissue-specific gene ablation.. Mech Dev.

[47] Uren AG, Mikkers H, Kool J, van der Weyden L, Lund AH (2009). A high-throughput splinkerette-PCR method for the isolation and sequencing of retroviral insertion sites.. Nat Protoc.

