# Supplementary figures and images for: piggyBac Transposon Somatic Mutagenesis with an Activated Reporter and Tracker (PB-SMART) for Genetic Screens in Mice
Source: PLoS One. 2011 Oct 21;6(10):e26650. doi: 10.1371/journal.pone.0026650 (PMC3198810; doi:10.1371/journal.pone.0026650)

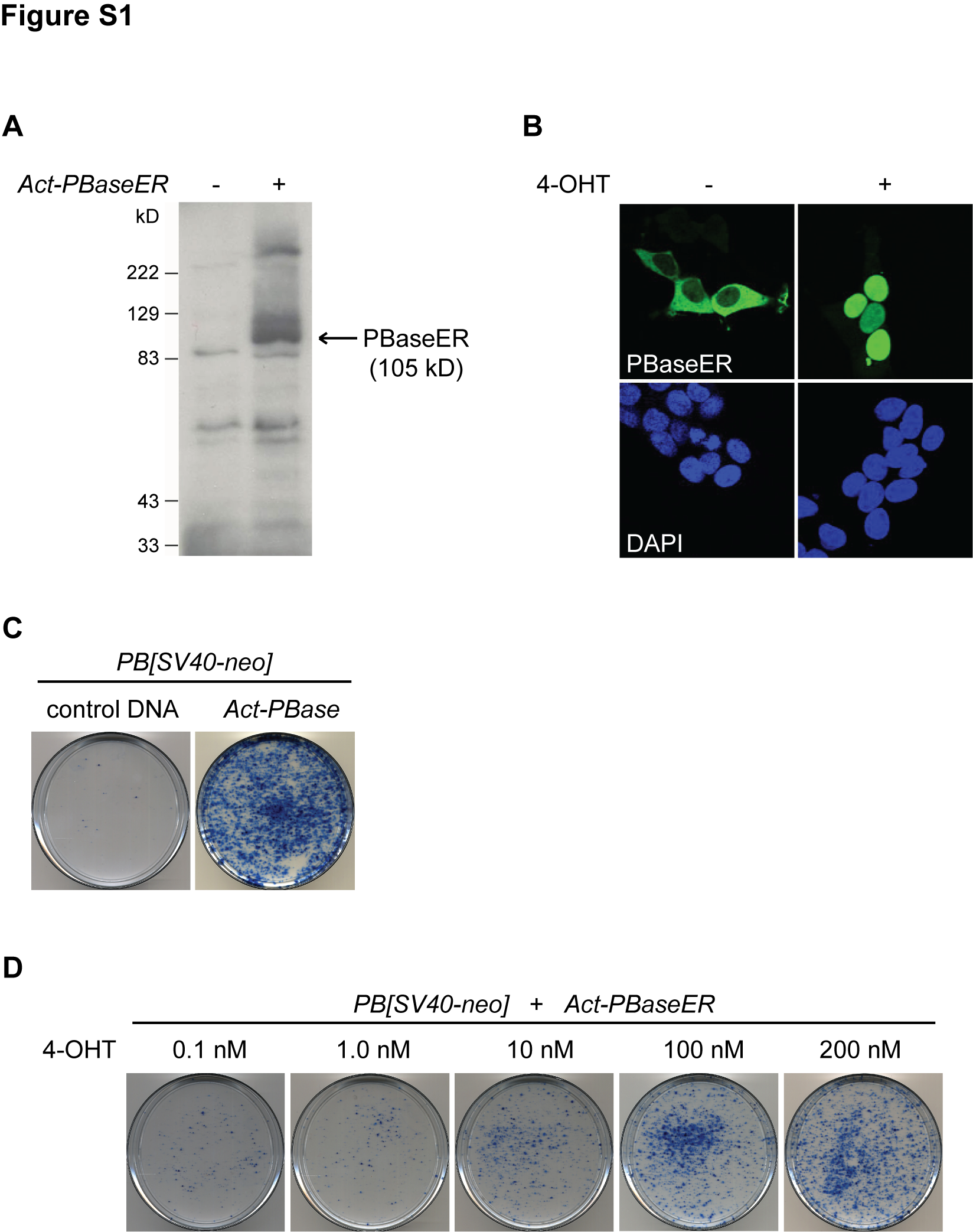

Supplement: Figure S1 — Tamoxifen-dependent translocation of PBaseERto the nucleus drives PB transposition in HEK 293 cells. (A) Act-PBaseER in transfected HEK 293 cells was detected by Western blotting using a polyclonal ERα antibody and displayed the expected size. (B) Immunofluorescent staining with ERα antibody revealed that PBaseER translocates to the nucleus after 24-hour 4-hydroxytamoxifen (4-OHT) treatment. (C) HEK 293 cells co-transfected with circular plasmids carrying PB with a neomycin resistance cassette (PB[SV40-neo]) and Act-PBase form G418-resistant clones after drug selection for two weeks, indicative of PBase-mediated transposition activity. (D) 4-OHT treatment is required for PBaseER-mediated transposon insertion. Compared to Act-PBase, Act-PBaseER mediates transposition at a lower efficiency. (TIF) [file pone.0026650.s001.tif]
