# Supplementary material for: piggyBac Transposon Somatic Mutagenesis with an Activated Reporter and Tracker (PB-SMART) for Genetic Screens in Mice
Source: PLoS One. 2011 Oct 21;6(10):e26650. doi: 10.1371/journal.pone.0026650 (PMC3198810; doi:10.1371/journal.pone.0026650)
Supplement: Table S1 — Primers used in this work for genotyping transgenic lines, analyzing PB excision, analyzing PB copy number, analyzing relative transcript levels, and mapping PB insertion sites. (DOC) [file pone.0026650.s002.doc]

**Table S1. Primers used in this work.**

| **Primers for genotyping transgenic lines** | | | |
| --- | --- | --- | --- |
| **Line(s)** | **Primer Name** | **Sequence** | **Product Size** |
| *Luc-PB[mut]* | LucPBLF | 5'-TGA ATACGATTTTGTGCCAG-3' | 1.4 kb |
|  | LucR | 5'-GGATCCTTATCGATTTTACC-3' |  |
| *PB[mut-RFP]* | PBRID1 | 5'-TGTTTTATCGGTCTGTATATCGAGG-3' | 160 bp |
|  | PBRID2B | 5'-AAAGTTTTGTTACTTTATAGAAGAAATTTTGAG-3' |  |
| *Act-PBase(ER)* | CAGF | 5'-GCAACGTGCTGGTTATTGTG-3' | 150 bp |
|  | PBASEER2 | 5'-CTGTCAGAATCCTCACCAAC-3' |  |
| *LSL-PBase* | CAGF | 5'-GCAACGTGCTGGTTATTGTG-3' | 250 bp |
|  | STOPR | 5'-GGTTTCTTCGGTGGAAACAA-3' |  |
| *All Cre lines* | CREF | 5'-CCGGGCTGCCACGACCAA-3' | 450 bp |
|  | CRER | 5'-GGCGCGGCAACACCATTTTT-3' |  |
| **Primers for analyzing PB excision frequency in transgenics by qPCR** | | | |
| **Purpose** | **Primer Name** | **Sequence** |  |
| Excision | LUCTTAAF1 | 5'-CCAGGGATTTCAGTCGATGT-3' |  |
|  | LUCTTAAR1 | 5'-GGACTCTGGCACAAAATCGT-3' |  |
| Control | ACTGF1 | 5'-CCCCAACACACCTAGCAAAT-3' |  |
|  | ACTGR1 | 5'-ACTGCCCCATTCAATGTCTC-3' |  |
| **Primers for analyzing PB copy number in transgenics by qPCR** | | | |
| **Purpose** | **Primer Name** | **Sequence** |  |
| Copy Number | PBLRTF2 | 5'-TCACGCGGTCGTTATAGTTCAA-3' |  |
|  | PBLRTR2 | 5'-CCGTGAGGCGTGCTTGTC-3' |  |
| Control | ACTGF1 | 5'-CCCCAACACACCTAGCAAAT-3' |  |
|  | ACTGR1 | 5'-ACTGCCCCATTCAATGTCTC-3' |  |
| **Primers for real-time PCR analysis of relative transcript levels** | | | |
| **Target Gene** | **Primer Name** | **Sequence** |  |
| *Actb* | ACTBFW | 5'-CCCTAAGGCCAACCGTGAA-3' |  |
|  | ACTBRV | 5'-CAGCCTGGATGGCTACGTACATG-3' |  |
| *Fli1* | FLI1F | 5'-CCCAGCCAGATCCTTATCAGATC-3' |  |
|  | FLI1R | 5'-CGGACAGTAGTTCCAGGAGAAACT-3' |  |
| *Gli2* | GLI2F | 5'-CTGAAGGATTCCTGCTCGTG-3' |  |
|  | GLI2R | 5'-GCCCCCTGTATTGTTGAAGT-3' |  |
| *Hprt* | HPRTF | 5'-TCCTCCTCAGACCGCTTTT-3' |  |
|  | HPRTR | 3'-CCTGGTTCATCATCGCTAATC-3' |  |
| *Mitf* | MITFF3 | 5'-AACCGACAGAAGAAGCTGGA-3' |  |
|  | MITFR3 | 5'-GGTGGATGGGATAAGGGAAA-3' |  |
| **Purpose** | **Primer Name** | **Sequence** | **Product Size** |
| Excision | TRN-131 | 5'-GTCGGACAATATCAAGTCGATGAGCG-3' | 212 bp |
|  | TRN-81 | 5'-GGCCACAATGTGGTTTTTGTCAAACGAAG-3' |  |
| Concatamer | LR2 | 5'-GGCCACAATGTGGTTTTTGTCAAACGAAG-3' | 700 bp |
|  | TRN-81 | 5'-GGCCACAATGTGGTTTTTGTCAAACGAAG-3' |  |
| **Oligonucleotides for LM-PCR identification of insertion sites** | | | |
|  | **Oligo Name** | **Sequence** |  |
|  | SPLNKL | 5'-CGAAGAGTAACCGTTGCTAGGAGAGACCGTGGCTGAATGAGACTG  GTGTCGACTCCGCTTAAGGGAC-3' | |
|  | LINKER- | 5'-PO_4_-GATCGTCCCTTAAGCGGAG-spacer-C3-3' |  |
|  | LINKER-3 | 5'-PO_4_-CGGTCCCTTAAGCGGAG-spacer-C3-3’ |  |
|  | SP1 | 5'-CGAAGAGTAACCGTTGCTAGGAGAGACC-3' |  |
|  | SP2 | 5'-GTGGCTGAATGAGACTGGTGTCGAC-3' |  |
|  | PB5-1 (PBR) | 5'-TAAATAAACCTCGATATACAGACCGATAAA-3' |  |
|  | PB5-2 (PBR) | 5'-ATATACAGACCGATAAAACACATGCGTCAA-3' |  |
|  | PB3-1 (PBL) | 5'-CAAAATCAGTGACACTTACCGCATTGACAA-3' |  |
|  | PB3-2 (PBL) | 5'-CTTACCGCATTGACAAGCACGCCTCACGGG-3' |  |
